# Multiple organ dysfunction and systemic inflammation after spinal cord injury: a complex relationship

**DOI:** 10.1186/s12974-016-0736-y

**Published:** 2016-10-06

**Authors:** Xin Sun, Zachary B. Jones, Xiao-ming Chen, Libing Zhou, Kwok-Fai So, Yi Ren

**Affiliations:** 1 Guangdong-Hong Kong-Macau Institute of CNS Regeneration, Joint International Research Laboratory of CNS Regeneration Ministry of Education of PRC, Guangdong Medical Key Laboratory of Brain Function and Diseases, Jinan University, Guangzhou, China; 2 Co-innovation Center of Neuroregeneration, Nantong, China; 3 Department of Biomedical Sciences, Florida State University College of Medicine, Tallahassee, FL USA; 4 Institute of Inflammation and Diseases, The First Affiliated Hospital of Wenzhou Medical University, Wenzhou, China

**Keywords:** Spinal cord injury, Multiple organ dysfunction, Systemic inflammatory response syndrome, Immune depression syndrome, Post-injury infection

## Abstract

Spinal cord injury (SCI) is a devastating event that results in significant physical disabilities for affected individuals. Apart from local injury within the spinal cord, SCI patients develop a variety of complications characterized by multiple organ dysfunction or failure. These disorders, such as neurogenic pain, depression, lung injury, cardiovascular disease, liver damage, kidney dysfunction, urinary tract infection, and increased susceptibility to pathogen infection, are common in injured patients, hinder functional recovery, and can even be life threatening. Multiple lines of evidence point to pathological connections emanating from the injured spinal cord, post-injury systemic inflammation, and immune suppression as important multifactorial mechanisms underlying post-SCI complications. SCI triggers systemic inflammatory responses marked by increased circulation of immune cells and pro-inflammatory mediators, which result in the infiltration of inflammatory cells into secondary organs and persistence of an inflammatory microenvironment that contributes to organ dysfunction. SCI also induces immune deficiency through immune organ dysfunction, resulting in impaired responsiveness to pathogen infection. In this review, we summarize current evidence demonstrating the relevance of inflammatory conditions and immune suppression in several complications frequently seen following SCI. In addition, we highlight the potential pathways by which inflammatory and immune cues contribute to multiple organ failure and dysfunction and discuss current anti-inflammatory approaches used to alleviate post-SCI complications. A comprehensive review of this literature may provide new insights into therapeutic strategies against complications after SCI by targeting systemic inflammation.

## Background

Spinal cord injury (SCI) causes disastrous damage to patients. While intraspinal infections, ischemia, and tumors can give rise to non-traumatic SCI, the majority of SCI is caused by physical trauma to the spine from sports injuries, car accidents, falls, and gunshots. Traumatic SCI induced by contusion of the spinal cord has a two-phase pathology characterized by primary and secondary injuries [1]. Primary injury can result from physical compression of the spinal cord, stretching of the nervous tissue, or disruption of local blood supply. This trauma causes the spine to deform and narrows the spinal canal, leading to dramatic changes in spinal cord volume. Mechanical damage may also impact blood vessels, immediately inducing intraspinal hemorrhage or reducing blood supply. Pathologically, primary injury occurs in a short window of time and within a limited area; represents direct damage of neurons, glial, or endothelial cells due to mechanical insults; and is characterized by hemorrhage, edema, and ischemia.

Secondary injury is a compilation of complex events subsequent to the initial trauma that develops minutes to weeks after SCI. Several mechanisms underlie the pathogenesis of secondary injury, including neurodegeneration, gliosis, and inflammation. Progressive enlargement of the affected regions exacerbates dysfunction by inducing apoptosis in nearby intact neural tissues. Proliferation or hypertrophy of activated glial cells, such as microglia and astrocytes, leads to the formation of glial scars. The inflammatory microenvironment following SCI is mediated by activated microglia and astrocytes, and infiltrating macrophages greatly contribute to the progression of secondary injury [2–6]. Angiogenic responses and remodeling of vascular structure also contribute to the development of secondary injury. It is well-acknowledged that effective restraint of secondary injury plays a fundamental role in minimizing neurodegeneration and significantly improves functional recovery after SCI. As such, much effort has been devoted to developing strategies that ameliorate secondary injury and facilitate neuroregeneration, such as inhibiting inflammation, blocking endogenous axon growth inhibitors (Nogo and CSPG), and reducing glial scars.

The functional consequences of SCI are largely determined by the level and completeness of the injury. First, the effect of SCI on loss of motor and non-motor function depends on the site of the injury. Nerves controlled by spinal cord segments below the injury site often lose their connections, and thus, the body-brain communication through descending motor pathways and ascending sensory pathways is disrupted in SCI. Due to the anatomical organization of the spinal cord, more rostral injuries are associated with greater levels of functional impairment. Injuries to cervical segments of the spinal cord result in the loss of motor and/or sensory function in the upper and lower limbs and often the trunk (tetraplegia or quadriplegia), whereas injuries to thoracic, lumbar, or sacral segments generally spare upper limb function and involve the lower limbs and trunk to varying degrees (paraplegia) [7]. Second, the extent or completeness of the injury is another determinant of SCI-elicited dysfunction. At the most basic level, SCIs can be classified as either complete or incomplete. Complete SCI represents an absence of motor and sensory function in S4–S5 segments (i.e., no sacral sparing), whereas in incomplete SCI there is preservation of some motor and/or sensory function below the level of injury. The incompleteness of SCI can be further divided according to the American Spinal Injury Association Impairment Scale, which grades injuries based on the amount of function preserved in patients [7].

## Multiple organ dysfunction after SCI

Beyond impairments to sensation and voluntary movement, SCI disturbs the autonomic nervous system and induces dysfunction or failure in multiple organs because of the critical role of the spinal cord in coordinating bodily functions [8]. Short- and long-term complications following SCI can occur in the nervous system (such as neurogenic pain and depression), lungs (such as pulmonary edema and respiratory failure), cardiovascular system (such as orthostatic hypotension and autonomic dysreflexia), spleen (such as splenic atrophy and leukopenia), urinary system (such as neurogenic bladder, kidney damage, and urinary tract infection), skeletal muscle (such as muscle spasticity and atrophy), bone and soft tissue (such as osteoporosis and heterotopic ossification), and skin (pressure sores) and include sexual dysfunction, hepatic pathology, neurogenic bowel dysfunction, syringomyelia, and increased susceptibility to infection. Some complications are high risk factors of mortality in SCI patients, e.g., liver, lung, and kidney damage, and therefore, therapeutic interventions that ameliorate post-SCI complications may be as important for prolonging life expectancy and improving life quality as those interventions that promote neuroregeneration and motor function recovery.

Multiple organ dysfunction after SCI is under complex regulation by multiple components. Cranial nerves emanating from brainstem areas (such as the pons and medulla) control the functions of multiple organs, and brainstem reflexes were reported to be changed in human patients with SCI [9]. This suggests a complex relationship between multiple organ dysfunction, the injured spinal cord, and altered brainstem activity. While much consideration should be given to the brainstem’s role in multiple organ dysfunction following SCI, this review focuses on the contributive roles of inflammation and immunity in these systemic complications.

## Systemic inflammation following SCI

The local inflammatory microenvironment within the injured spinal cord is a collection of degenerating neurons, degraded myelin sheath, damaged endothelial cells, and activated glial and infiltrating cells, and this microenvironment produces various kinds of pro-inflammatory mediators [10]. In addition to this intraspinal inflammation, SCI can trigger systemic inflammatory response syndrome (SIRS), a life-threatening condition which can affect distal organs [11–15]. Epidemiological analyses have revealed a functional link between systemic inflammation and pathogeneses of post-injury complications: SIRS-positive SCI patients have higher injury severity and a higher incidence of complications than do SIRS-negative patients [16]. Many other factors, such as dysregulation of the neuroendocrine system and altered neuroimmune regulation, are important determinants of the onset and progression of post-SCI systemic inflammation. For instance, SCI activates the hypothalamic-pituitary-adrenal axis, leading to increased macrophage migration inhibitory factor production by the pituitary gland [17]. Macrophage migration inhibitory factor is extensively involved in systemic inflammation, suggesting that SCI-elicited neuroendocrine changes contribute to the progression of systemic inflammation. Chronic activation of microglia, the neuroimmune cells of the central nervous system, occurs in the hippocampus and cerebral cortex after SCI, indicating neuroimmune dysregulation is involved in systemic inflammation following SCI [18].

## Immune suppression induced by SCI

Another consequence of post-injury spinal cord-immune system interplay is SCI-induced immune depression syndrome (SCI-IDS) which is due, at least in part, to dysregulation of the sympathetic nervous system and immune organ dysfunction [19–21]. SCI can lead to sympathetic nervous system dysfunction directly by severing thoracolumbar spinal cord projections to sympathetic ganglion or indirectly by disrupting supraspinal control through the hypothalamic-pituitary-adrenal axis. SCI-IDS, indicated by the loss of splenocytes and leukopenia, is a putative self-defense mechanism that lowers potential autoimmunity to self-antigens produced by damage in the central nervous system [22, 23]. In contrast to this protective effect, accumulating evidence has shown that SCI-IDS worsens neurological conditions and impairs the functional recovery of SCI patients. Riegger et al. reported significant decreases in the number of circulating cells involved in both innate and adaptive immunity in the acute phase following rat SCI [24]. Similar observations were made in a pilot study involving 16 SCI patients and ten healthy controls: decreased monocytes, T lymphocytes, and B lymphocytes, but not granulocytes, were observed in peripheral blood within 24 h after SCI [25]. SCI-IDS has important clinical relevance, as SCI patients display increased susceptibility to various infections (e.g., pneumonia and wound infections) [26] and poor functional recovery [27]. Autonomic dysreflexia and the expansion of myeloid-derived suppressor cells following SCI may play causal roles in SCI-IDS [28, 29], though the underlying mechanisms of SCI-IDS are still largely unknown.

## Role of inflammation and immunity in post-SCI complications

### Nervous system

Hyperesthesia, i.e., increased sensitivity to somatosensory stimuli, frequently occurs after SCI. Many brain regions that participate in nociception, e.g., the thalamus and nucleus accumbens, undergo neuronal changes following SCI [30]. For example, spinal cord contusion results in increased responsiveness of thalamic neurons to somatosensory stimuli [31], and this effect is partially mediated by upregulation of sodium channel Nav1.3 [32]. In addition, the inflammatory response plays an important role in chronic pain after SCI [33]. Progressively activated microglia have been observed in the thalamus not only in the acute phase (several days after SCI) but also in the chronic phase (several weeks after SCI) [34]. Chemokines CCL2 and CCL3, key players in neuropathic pain, were detected in the thalamus and hippocampus in the chronic phase after severe SCI [35, 36]. Chemokine CCL21, induced in lumbar dorsal horn neurons after SCI, may mediate remote activation of cerebral microglia. Neutralization of CCL21 suppressed microglia activation and subsequent hyperexcitability of thalamic neurons [37, 38]. Meanwhile, upregulation of proteins involved in the cell cycle (e.g., cyclin D1) has been associated with microglia activation, and thus, ablation of cell cycle signaling could significantly reduce neuroinflammation and ameliorate motor dysfunction and post-traumatic hyperesthesia [34, 39]. Decreased expression of pro-inflammatory cytokines by progesterone, a neuroactive steroid, also alleviated chronic pain after SCI [40].

Cognitive impairment is associated with extensive cerebral inflammation after SCI [41]. Mice undergoing traumatic SCI exhibited impaired learning and memory associated with elevated neuroinflammation in the hippocampus and cortex, whereas interventions that attenuated inflammatory responses facilitated cognitive function recovery [18, 42, 43]. Nonetheless, a better understanding of the methods and consequences of efficient inhibition of inflammation is necessary to yield significant improvements in human cognitive function following SCI.

Depression is another common complication in SCI patients, especially those with an early onset [44, 45]. To date, the relationship between depression and neuroinflammation after SCI remains elusive. It has been noted that the serum concentration of corticosterone, an inducer of depressive-like phenotypes in animal models, was elevated shortly after SCI and remained so for at least 1 month after experimental SCI in rats [11]. Wu et al. also reported depressive-like behaviors in mice with spinal cord contusion [18]. In a rat model of SCI, high levels of pro-inflammatory cytokines were associated with comorbidity of depression and anxiety, and there was no correlation between these comorbidities and trauma severity [46]. These results help to explain how activation of microglia and astrocytes may contribute to psychiatric complications; furthermore, they underscore the therapeutic potential of targeting these cells. Recently, a randomized clinical trial revealed the effectiveness of targeting inflammation to improve mood in SCI patients by reducing IL-1β and increasing the levels of a neuroactive compound involved in the kynurenine pathway [47]. Taken together, brain dysfunction and neurodegeneration, common complications of SCI, may be closely related to cerebral inflammation, characterized by elevated pro-inflammatory cytokines and activation of microglia and astrocytes. Although a better understanding of this relationship is needed, targeting inflammation in the brain may serve as an important therapeutic approach to improve the overall quality of life for SCI patients.

### Lung

Pulmonary complications, such as respiratory failure and pulmonary infection, are quite common in SCI patients and largely contribute to morbidity and mortality in these affected individuals [48, 49]. SCI patients have an increased likelihood of developing pulmonary dysfunction, including acute respiratory distress syndrome and acute lung injury [50]. While impaired vascular and muscular functions could explain, at least in part, the development of pulmonary complications, the fact that patients with lower thoracic SCI still develop pneumonia and respiratory dysfunction suggests that the mechanisms involved in respiratory complications induced by SCI are complex [51]. Insights into lung inflammation after SCI may inspire the development and adoption of novel strategies to treat pulmonary complications.

The lungs are a major target of SCI-induced acute inflammation. Using a rat model of spinal cord compression, Gris et al. reported significant neutrophil activation and lung tissue invasion several hours after SCI, and substantial macrophage infiltration was also found in the lungs 3 days post injury [52]. Notably, the early onset of pulmonary inflammation is consistent with the development of lung dysfunction in the early stage of SCI [51]. Two cross-sectional studies involving chronic SCI patients unveiled a correlation between pulmonary inflammation and dysfunction: SCI patients with higher levels of inflammation markers IL-6 and C-reactive protein had reduced lung volume measurements [53, 54]. Consequently, pharmaceutical interventions that decrease systemic inflammation in the lungs may alleviate pulmonary dysfunction [55–57]. Resveratrol, an anti-inflammatory agent [58] which exhibits neuroprotective effects in spinal cord injuries [59–61], successfully reduced pulmonary infiltration of neutrophils and the production of pro-inflammatory mediators, suppressed NF-κB activation, and ameliorated pulmonary edema [62]. This suggests that antagonizing inflammation in the lungs promotes recovery of impaired pulmonary functions after SCI. Indeed, a small-molecule agonist of dopamine D1 receptor [63] effectively attenuated pulmonary edema and lung damage following SCI [64] via inhibition of NLRP3 inflammasome activation.

### Cardiovascular system

Patients with spinal cord damage often suffer from cardiovascular disease, a leading cause of death for these individuals [65]. Following SCI, autonomic nervous system impairment results in blood pressure and heart rate dysregulation [66]. Autonomic dysreflexia, characterized by acute hypertension after afferent stimulation, is frequently seen in SCI patients, especially those with high-level injuries [67]. The development of autonomic dysreflexia in SCI mice has also been demonstrated [68]. Blockade of inflammation-related receptors, e.g., CD11d, CXCR1, and CXCR2, attenuated the development of autonomic dysreflexia after SCI [69–71], highlighting anti-inflammatory treatments as potential therapeutics against cardiovascular dysfunction post injury.

### Liver

While few investigations have examined the effect of SCI on liver function, Sipski and colleagues have reported hepatic abnormalities in chronic SCI patients [72]. It is reasonable to suspect that liver dysfunction can be associated with spinal cord trauma, as the liver plays an essential role in the metabolic dysfunction commonly observed after SCI. Indeed, animal studies have revealed that traumatic injury to the spinal cord triggers neutrophil infiltration, macrophage activation, and the expression of pro-inflammatory cytokines and chemokines in the liver [73, 74]. This inflammation appears as early as 30 min after injury [75], and its severity is correlated with lesion level [76]. Of note, considerable lipid accumulation has been detected in rodent livers after SCI [77]. Given the pro-inflammatory and cytotoxic effects of myelin-laden macrophages associated with lipid accumulation in the injured spinal cord [2–4, 78], macrophage-mediated inflammation may substantially contribute to hepatic dysfunction after SCI.

### Spleen

The spleen—innervated by the autonomic nervous system and controlled by the high thoracic spinal cord—is an important lymphoid organ and source of infiltrating monocytes in the injured spinal cord. In a mouse SCI model, significant dysfunction of the spleen was observed following high thoracic SCI (T3 section), whereas lower thoracic SCI (T9 section) preserved the majority of splenic function. Spleen dysfunction after T3 SCI was indicated by splenic atrophy with reduced spleen size, decreased splenic leukocyte numbers, and increased splenic norepinephrine [21, 28]. SCI mice challenged with viral infection show impaired protective immune responses and decreased survival, and these outcomes were associated with deficient CD4+ and CD8+ T cell functions, suppressed activation of macrophages, and deficient primary antibody response [79–81]. This suggests that splenic dysfunction may largely contribute to immune suppression in SCI patients. Notably, post-SCI mRNA levels of pro-inflammatory cytokines IL-17 and IL-23 were upregulated in rat splenic tissue through STAT3 signaling [82], and thus, crosstalk between the peripheral spleen and injured spinal cord may be mediated by neuroinflammation.

### Gastrointestinal tract

Gastrointestinal dysfunction, e.g., severe constipation, difficulty with evacuation, painful defecation or incontinence, is a common complication following SCI and is quite restrictive for patients, limiting their diet and outdoor activity [83]. Although the function of the gastrointestinal tract is primarily determined by its own intrinsic nervous system and autonomic control from the brainstem, spinal cord trauma may damage the neuronal control of gastrointestinal sensory and motor functions, resulting in neurogenic bowel dysfunction (NBD). As approximately half of SCI patients suffer from moderate to severe NBD [84], it seems that abnormal bowel function exerts a highly negative impact on life quality for SCI patients [85, 86]. The clinical manifestations of NBD with SCI include decreased colonic motility, prolonged bowel transit time, and anorectal dysfunction [87]. Although inflammation has been observed in colonic lesions of SCI patients [88], the relationship between systemic inflammation and NBD largely remains unclear. Utilizing a rat model of NBD after SCI, Guo et al. reported that upregulation of neuronal nitric oxide synthase contributed to colonic dysfunction [89], thus establishing inflammation as a potential target for alleviating post-SCI NBD.

### Urinary system

SCI may impair supraspinal control of the bladder and result in neurogenic bladder, characterized by dysfunction in bladder storage and emptying [90–92]. Accordingly, SCI patients have an increased risk of developing urinary tract infections and renal damage, both of which can be life-threatening [93–96]. Besides the direct loss of neuronal input after injury, inflammation has been implicated in the pathogenesis of urinary system dysfunction in SCI patients. Such inflammation may include infiltration of immune cells, production of pro-inflammatory cytokines (IL-1β, IL-6, and TNF-α), upregulation of myeloperoxidase, inducible nitric oxide synthase and cyclooxygenase-2, and activation of NF-κB [52, 94, 97–100]. Many potential therapeutic approaches have been investigated to alleviate bladder dysfunction and kidney damage. Blockade of pro-inflammatory integrin signaling by antibodies targeting CD11d or CD49d has been shown to reduce kidney inflammation induced by SCI [55, 57]. Application of antioxidant vitamin C and activation of adrenoreceptors protected rat kidneys from SCI-induced damage by suppressing NF-κB signaling and pro-inflammatory cytokine expression [94, 97]. Oral administration of anti-inflammatory small-molecule S-nitrosoglutathione can promote recovery of neurogenic bladder via inhibition of inflammatory responses [98]. Oxidants such as dantrolene and quercetin are able to ameliorate urinary bladder lesions after SCI by decreasing bladder hemorrhage and immune cell infiltration [101, 102]. The aforementioned results are a promising sign for the treatment of SCI-induced urinary system dysfunction.

### Skeletal muscle

Skeletal muscles controlled by the spinal cord below the injured area become paralyzed and develop atrophy after SCI [103, 104]. Physiological tests have revealed numerous changes in the properties of disabled muscles from SCI patients, including decreased muscle cross-sectional area, reduced muscle mass, increased susceptibility to fatigue, and an increased proportion of fast glycolytic muscular fibers [105–107]. While it is well-known that paralysis-induced disuse is the primary cause of muscular atrophy, emerging evidence suggests that the involvement of inflammation in post-SCI muscle dysfunction may not be trivial. Muscular inflammation can be observed in the acute phase of SCI even before obvious muscle atrophy is seen [108]. In long-term SCI, muscle atrophy is associated with a significant elevation of inflammatory mediators (e.g., IL-1β, IL-6, and TNF-α) and activation of NF-κB signaling [109, 110], a key regulator of the inflammatory state in muscle atrophy [111]. In a mouse transection SCI model, administration of glutamine—which is extensively used to improve clinical outcomes—has been shown to decrease pro-inflammatory cytokine expressions (IL-6 and TNF-α), attenuate loss of myofibrillar proteins in muscle, and mitigate muscular fatigability [112]. Therefore, administration of anti-inflammatory agents may serve as a promising therapeutic approach to accelerate muscular function recovery after SCI.

### Bone

Osteoporosis, characterized by the loss of bone mineral density (BMD), is a well-defined consequence of SCI [113, 114]. The distal femur, proximal tibia, and distal boney sites at sublesional levels are the most susceptible to BMD loss [115, 116]. The decrease in BMD is progressive after SCI and increases patients’ risk of fracture [117], and many factors contribute to the pathogenesis of osteoporosis following SCI. In addition to deficiencies in neuronal control, hormonal regulation, and vascular function [118–120], the inflammatory microenvironment in bone also mediates osteoclast differentiation and bone loss [121–123]. Elevated levels of IL-6 have been detected in conditioned medium from bone marrow cultures of SCI patients [124]. Similarly, significant increases in serum IL-6 (approximately 2.5-fold compared to sham) and *IL-6* mRNA (approximately sixfold compared to sham) were observed in the femurs of rats post-SCI [125]. IL-6 signaling stimulates osteoclast progenitors to differentiate into osteoclasts [126–129] and suppresses osteoblast differentiation [130, 131], resulting in bone resorption. Blockade of IL-6 by its neutralizing antibody inhibited osteoclast-like cell formation in SCI patient-derived bone marrow cultures [124]. In addition, resveratrol significantly suppressed IL-6 expression in femurs and reduced osteoclastogenesis in SCI rats [125]. Hence, inflammatory mediators are active players in osteoporosis after SCI and may be important anti-osteoporotic targets.

### Soft tissue

Neurogenic heterotopic ossification (HO), a process by which new bone formation occurs outside of the skeleton and preferentially around soft tissues, is an irreversible complication of SCI more frequently seen in young patients [132–135]. Cervical and thoracic SCIs induce more HO than do lumbar injuries, and the hip joint is the major ossified area. Regulation of HO development is multifactorial [136–138], and the inflammatory response is an important contributing factor in the early stage of HO. Among the multiple therapeutic choices, non-steroidal anti-inflammatory drugs are effective prophylactic treatments against HO when administered soon after SCI [134, 139–143]. Estrores and colleagues reported that increased levels of C-reactive protein, a commonly used marker of acute inflammation in SCI [144–146], were associated with early occurrence of HO and the concentration of C-reactive protein declined when HO symptoms were alleviated in later stages [147]. The lack of animal models that reproduce the clinical features of HO observed in SCI patients has long hindered the study of underlying HO mechanisms; however, in 2015 a well-characterized mouse HO model showed that phagocytic macrophages play a critical role in driving the development of HO [148]. This study highlighted the impact of the inflammatory microenvironment on HO and may benefit the discovery of novel anti-inflammatory treatments against HO.

### Syringomyelia

Syringomyelia is a relatively rare sequela defined by cavity formation in the injured spinal cord via enlargement of the central canal [149]. Syringomyelia induces devastating symptoms including muscle weakness, loss of sensitivity, stiffness, and pain. In SCI rats, induced inflammatory conditions exacerbated syrinx formation, indicating a potential role of inflammation in the pathogenesis of syringomyelia [150].

## Conclusions

**Fig. 1:**
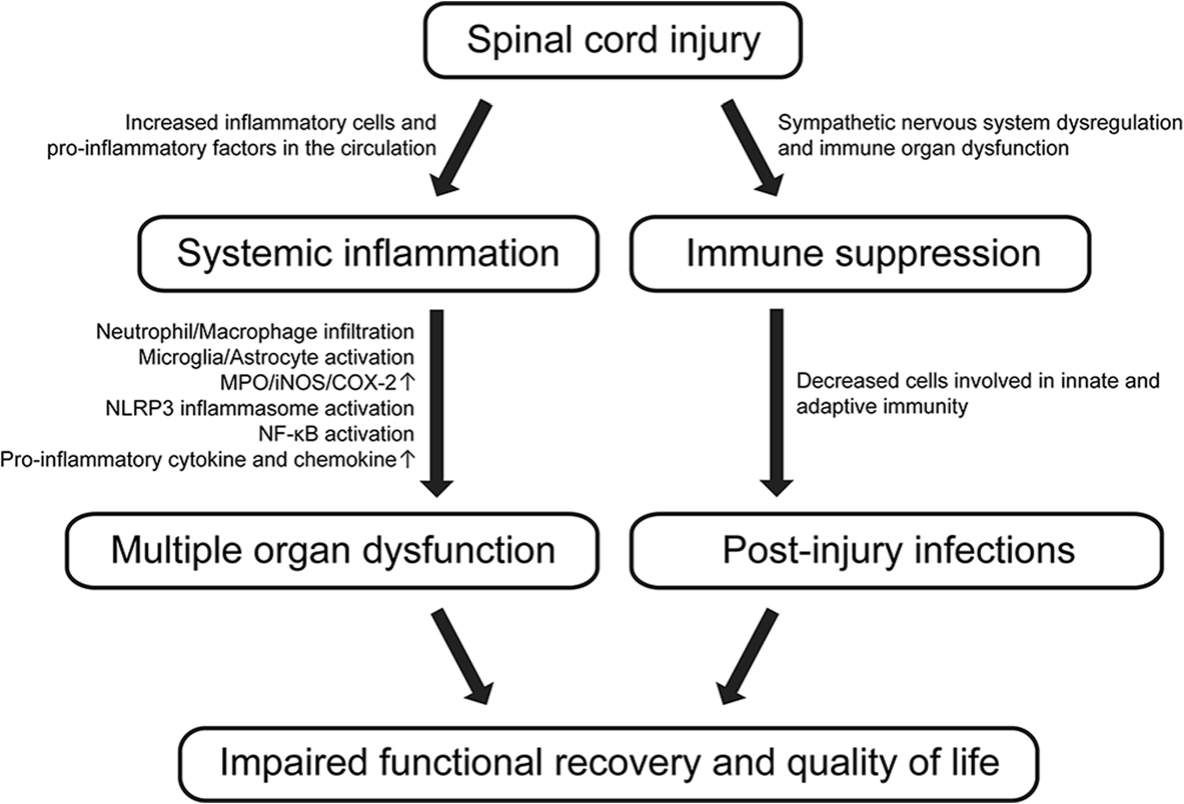
Schematic diagram of systemic inflammation- and immune depression-associated multiple organ dysfunction following SCI. SCI triggers an acute increase of inflammatory cells (such as neutrophils and macrophages) in the circulation and elevates serum concentrations of pro-inflammatory mediators. Subsequent infiltration of inflammatory cells from the blood into secondary organs initiates a series of events that mediate inflammatory responses in these organs. Activation of resident immune cells (microglia) in the brain is also found after SCI. SCI itself interrupts innervation of immune organs by the sympathetic nervous system, causing immune depression syndrome. Suppressed immunity leads to an increased susceptibility of the whole body to post-injury pathogen infections through decreased immune cell quantities (such as monocytes, T cells, and B cells)

Post-SCI multiple organ dysfunction is influenced by multifactorial mechanisms, and the extent to which systemic inflammation and immune depression contribute to SCI-associated complications is still an open question. Nevertheless, a growing body of evidence demonstrates the involvement of inflammatory conditions in the damage or dysfunction of multiple organ systems secondary to SCI. Systemic inflammatory responses following SCI induce infiltration of inflammatory cells into secondary tissues, activation of resident immune cells, and stimulation of pro-inflammatory cytokine production, all of which contribute to the pathogenesis of multiple organ dysfunction after SCI. Meanwhile, immune suppression subsequent to SCI significantly increases susceptibility to post-injury infection due to impaired innate and adaptive immunity in SCI patients, leading to worsened multiple organ damage and mortality. Therefore, inflammation and immunity not only contribute to the progression of intraspinal injury but also are important determinants of multiple organ dysfunction after SCI (Fig. 1).

Many anti-inflammatory strategies that attempt to ameliorate local intraspinal inflammation and promote neural tissue repair may find their true value in alleviating dysfunction in multiple organs secondary to the injured spinal cord. Such therapeutics may include immunomodulators of inflammation-associated pathways, e.g., estrogen, IL-33, IL-37, and adiponectin signaling pathways [151–154]. Small-molecule agonists or antagonists and blocking antibodies that specifically recognize and deactivate a variety of receptors involved in transduction of inflammatory signals—e.g., interleukin receptors, toll-like receptors, integrins, and estrogen receptors—are promising tools to mitigate complications after SCI [55–57, 92, 155–158]. Intracellular components of inflammatory machinery, including enzymes and transcription factors, may also serve as therapeutic targets to resolve inflammation in multiple organs following SCI [159–164].

Co-application of anti-inflammatory strategies with other treatment approaches after SCI may provide a therapeutic benefit for patients, though there is a lack of human clinical trials employing such strategies. In the future, well-designed experimental studies utilizing reliable animal models are needed to better understand the detailed mechanisms of how post-SCI complications develop with systemic inflammation and suppressed immunity and to suggest effective immunoregulatory approaches to mitigate SCI-induced multiple organ dysfunction. Such studies should be taken into consideration with the ultimate goal of developing therapies to improve the total body health of SCI patients.

## Abbreviations

BMD: Bone mineral density
HO: Heterotopic ossification
NBD: Neurogenic bowel dysfunction
SCI: Spinal cord injury
SCI-IDS: SCI-induced immune depression syndrome
SIRS: Systemic inflammatory response syndrome
